# Urinary Neutrophil Gelatinase-Associated Lipocalin (NGAL) in Patients with Obstructive Sleep Apnea

**DOI:** 10.1371/journal.pone.0154503

**Published:** 2016-05-05

**Authors:** Manish R. Maski, Robert J. Thomas, S. Ananth Karumanchi, Samir M. Parikh

**Affiliations:** 1 Division of Nephrology/ Department of Medicine, Beth Israel Deaconess Medical Center and Harvard Medical School, Boston, Massachusetts, United States of America; 2 Division of Pulmonary, Critical Care, & Sleep Medicine/ Department of Medicine, Beth Israel Deaconess Medical Center and Harvard Medical School, Boston, Massachusetts, United States of America; 3 Department of Obstetrics and Gynecology, Beth Israel Deaconess Medical Center and Harvard Medical School, Boston, Massachusetts, United States of America; University of Sao Paulo Medical School, BRAZIL

## Abstract

**Background:**

Obstructive sleep apnea (OSA) is a well-established risk factor for hypertension and cardiovascular morbidity and mortality. More recently, OSA has been implicated as an independent risk factor for chronic kidney disease. Urinary neutrophil gelatinase-associated lipocalin (NGAL) is a well-accepted early biomarker of subclinical kidney tubular injury, preceding an increase in serum creatinine. The goal of this study was to determine if an association exists between OSA and increased urinary NGAL levels.

**Methods:**

We prospectively enrolled adult patients from the sleep clinic of an academic medical center. Each underwent polysomnography and submitted a urine specimen upon enrollment. We measured NGAL and creatinine levels on all urine samples before participants received treatment with continuous positive airway pressure (CPAP), and, in a subset of OSA patients, after CPAP therapy. We compared the urinary NGAL/creatinine ratio between untreated participants with and without OSA, and within a subset of 11 OSA patients also after CPAP therapy.

**Results:**

A total of 49 subjects were enrolled: 16 controls based on an apnea-hypopnea index (events with at least 4% oxygen desaturation; AHI-4%) <5 events/hour (mean AHI-4% = 0.59 +/- 0.60); 33 OSA patients based on an AHI-4% >5 events/hour (mean AHI-4% = 43.3 +/- 28.1). OSA patients had a higher mean body-mass index than the control group (36.58 +/- 11.02 kg/m^2^ vs. 26.81 +/- 6.55 kg/m^2^, respectively; p = 0.0005) and were more likely to be treated for hypertension (54.5% vs. 6.25% of group members, respectively; p = 0.0014). The groups were otherwise similar in demographics, and there was no difference in the number of diabetic subjects or in the mean serum creatinine concentration (control = 0.86 +/- 0.15 mg/dl, OSA = 0.87 +/- 0.19 mg/dl; p = 0.7956). We found no difference between the urinary NGAL-to-creatinine ratios among untreated OSA patients versus control subjects (median NGAL/creatinine = 6.34 ng/mg vs. 6.41 ng/mg, respectively; p = 0.4148). Furthermore, CPAP therapy did not affect the urinary NGAL-to-creatinine ratio (p = 0.7758 for two-tailed, paired t-test).

**Conclusions:**

In this prospective case-control study comparing patients with severe, hypoxic OSA to control subjects, all with normal serum creatinine, we found no difference between urinary levels of NGAL. Furthermore, CPAP therapy did not change these levels pre- and post-treatment.

## Introduction

Obstructive sleep apnea (OSA) has been estimated to affect 24% of middle-aged men and 9% of middle-aged women, with 9% and 4%, respectively, meeting criteria for moderate-severe OSA [1]. It is characterized by recurrent complete or partial collapse of the upper airway leading to hypoxia and hypercapnea, and results in large fluctuations in intrathoracic pressure and compensatory arousals to restore respiration. The prevalence of OSA is only expected to increase in parallel with the obesity epidemic, since obesity is the greatest risk factor for its development.

OSA is a well-established independent risk factor for hypertension, coronary artery disease, congestive heart failure, stroke, and death [2–4]. Furthermore, significant evidence has accumulated implicating OSA as a risk factor for the initiation and progression of chronic kidney disease (CKD), independent of the frequently occurring co-morbidities between the two including diabetes, hypertension, and obesity [5–10]. In the largest study to evaluate the independent association between OSA and CKD, *Lee*, *et al* [11] compared 4,674 newly-diagnosed adult OSA patients to 23,370 age- and sex-matched non-OSA patients. The two groups were followed for the occurrence of CKD diagnosis: after adjustment for numerous potentially confounding comorbidities, OSA patients demonstrated a 1.94-fold increase in the incidence of CKD and a 2.2-fold increase in the incidence of end-stage renal disease (ESRD) [11].

The mechanisms by which OSA may directly damage the kidneys remain incompletely understood. Clearly, OSA may indirectly worsen renal function via exacerbation of both hypertension [12] and glycemic control [13], two of the best-established risk factors for CKD. However, experimental data suggest OSA may directly produce end-organ injury via mechanisms including excess sympathetic nervous system activity [14,15], increased renin-angiotensin-aldosterone system activity [16,17], hypoxia/reoxygenation-induced formation of reactive oxygen species [18], endothelial dysfunction [19], inflammation [20], and perturbations in renal hemodynamics [5, 21–23].

In particular, hemodynamic studies in OSA patients have demonstrated reduced diurnal renal blood flow that improves after treatment with continuous positive airway pressure (CPAP) [5,22], as well as impaired renal arterial vasodilating capacity [21]. Furthermore, in a porcine model of OSA, marked renal hypoperfusion was observed after the application of each obstructive respiratory event, with a mean drop in renal blood flow of over 60% (from 190 +/- 24 ml/min to 70 +/- 20 ml/min; P<0.00001) [23].

We therefore hypothesized that repetitive renal hypoperfusion events caused by obstructive apneas throughout the night, and perhaps diurnal reduction in renal blood flow, may be responsible for subacute kidney injury that accumulates over time. We reasoned that recurrent renal hypoperfusion of hypoxemic blood during each obstructive apnea, followed by restoration of perfusion of normoxic blood between apneas, may mimic an “ischemia-reperfusion” type [24] of cumulative renal tubular epithelial cell injury. Moreover, we posited that such renal tubular epithelial cell injury would be present before an increase in the serum creatinine manifests.

To test this hypothesis, we prospectively enrolled newly-diagnosed OSA patients and unaffected subjects from our Sleep Disorders Clinic to assay their urine for neutrophil gelatinase-associated lipocalin (NGAL). NGAL has emerged as one of the most promising urinary biomarkers for the early detection and prediction of kidney injury, generally preceding a detectable increase in the serum creatinine [25]. NGAL is highly upregulated and released into the urine by injured renal tubular epithelial cells soon after experimental ischemia-reperfusion injury in mice [26]. Moreover, in several studies of patients undergoing cardiac surgery—characterized by renal ischemia-reperfusion—elevated urinary NGAL levels both preceded acute kidney injury (AKI) defined as an increase in serum creatinine, and were also associated with poor clinical outcomes [27–29].

## Methods

### Study Design and Oversight

This prospective, observational study was performed at the Beth Israel Deaconess Medical Center (BIDMC, Boston, MA), in accordance with all institutional policies and with approval of the hospital’s institutional review board, the BIDMC Committee on Clinical Investigations (CCI). The BIDMC CCI first approved this study (under protocol number 2008P000467) on 3/13/2009 and has renewed the protocol annually. All participants provided written informed consent upon enrollment.

### Study Participants and Clinical Data

Adult participants were recruited from the BIDMC Sleep Disorders Clinic over the period from 2009–2014. The only exclusion criterion was established stage 3 chronic kidney disease or higher, corresponding to an estimated glomerular filtration rate of less than 60 mL/min/1.73m^2^ by the abbreviated MDRD equation [30]. Each participant’s medical record was reviewed for the following clinical information: gender, age, self-identified race, body mass index (BMI; kg/m^2^), diagnoses of hypertension, diabetes mellitus, and other chronic medical conditions, current medications, recent clinic blood pressure measurements, and recent serum creatinine measurements.

Standard in-center polysomnography (PSG) was performed on all patients around the time of study enrollment. PSGs were scored using standard American Academy of Sleep Medicine criteria [31] by registered sleep technologists. An *apnea* was defined as cessation of airflow for at least 10 seconds and a *hypopnea* as an abnormal respiratory event lasting at least 10 seconds with at least a 30% reduction in airflow and at least a 4% oxygen desaturation [31]. The apnea-hypopnea index-4% (AHI-4%) was calculated by summing all recorded apneas and hypopneas and dividing by the total hours of sleep recorded, resulting in units of *number of apneas and hypopneas with at least 4% oxygen desaturation per hour*. Additional sleep metrics were available for most participants, including: the respiratory disturbance index (RDI), which accounts for all scored respiratory events regardless of oxygen desaturation, sleep efficiency defined as the percentage of time actually asleep while in bed, and the percentages of total sleep time spent in non-rapid eye movement sleep stages 1 through 3 (Stages N1% through N3%) and in rapid eye movement sleep (REM%). While the control subjects did not qualify for a diagnosis of OSA, most were having sleep-related complaints that prompted their PSG, usually excessive daytime sleepiness. The average duration of sleep-related symptoms was similar for both the control and the OSA groups at the time of enrollment (approximately 2 years).

A subset of OSA patients who were successfully treated with CPAP submitted post-treatment urine samples for analysis. *Successful CPAP treatment* was defined as at least 1 month of CPAP therapy titrated to achieve the lowest possible residual AHI-4%, with documented compliance of at least 4 hours of use per night.

### Measurement of Urinary Creatinine and Neutrophil Gelatinase-Associated Lipocalin

Each participant provided a clean-catch urine sample upon enrollment (and for a subset of participants, also after successful treatment with CPAP) between the hours of 9:00AM and 4:00PM. Collected urine samples were immediately transported to the laboratory, aliquoted, and stored at -80 C until analysis. Urinary Neutrophil Gelatinase-Associated Lipocalin (NGAL) concentrations were determined via quantitative sandwich enzyme-linked immunosorbent assay (ELISA) (R&D Systems, Inc., Minneapolis, MN). Urinary creatinine concentrations were determined by Jaffe reaction using a commercially available assay (R&D Systems, Inc., Minneapolis, MN).

### Statistical Analysis

Participant characteristics and clinical data are presented either as means +/- standard deviations or as number of patients (N) falling within a category. Characteristics between OSA and control groups were compared using independent samples t-tests or Fisher’s exact tests, as appropriate. Pearson’s correlation coefficients were used to determine the associations between mean arterial pressure (MAP) and BMI, and between urinary NGAL per creatinine (NGAL/Cr) and AHI-4%. The Mann-Whitney test was used to compare the urinary NGAL/Cr ratios between control and untreated OSA groups. Paired t-test (two-tailed) was used to compare the urinary NGAL per creatinine ratios before and after treatment with CPAP among 11 OSA patients. Statistical analyses were conducted with the use of GraphPad Prism software, version 6.0d (GraphPad Software, Inc.). Two-tailed P values of less than 0.05 were considered to indicate statistical significance.

## Results

### Patient Characteristics

A total of 49 subjects were prospectively enrolled in this study: 16 subjects fell into the control group based on their PSG demonstrating fewer than 5 hypoxic events per hour (as defined by AHI-4%); 33 subjects fell into the OSA group based on an AHI-4% of greater than 5 events per hour. The OSA group had a significantly higher mean BMI than the control group (36.58 +/- 11.02 kg/m^2^ versus 26.81 +/- 6.55 kg/m^2^, respectively; p = 0.0005) and was much more likely to be on one or more medications for hypertension (54.5% versus 6.25% of group members, respectively; p = 0.0014). (Table 1).

**Table 1:** Patient Characteristics. OSA = obstructive sleep apnea, M = male, F = female, Afr Amer = African American, BMI = body mass index, DM = diabetes mellitus, HTN = hypertension, SBP = systolic blood pressure, DBP = diastolic blood pressure, AHI-4% = apnea-hypopnea index of respiratory events per hour with at least 4% oxygen desaturation, RDI = respiratory disturbance index, O2 sat % = percentage oxygen saturation recorded, Stage N1% = percentage of total sleep in non-REM sleep stage I, Stage N2% = percentage of total sleep in non-REM sleep stage II, Stage N3% = percentage of total sleep in non-REM sleep stage III, REM% = percentage of total sleep in rapid eye movement (REM) sleep. Table shows mean (+/- standard deviation) or N = number of patients falling into category.

	Control	OSA Untreated	P-value
N	16	33	
Sex (M/F)	6M / 10F	20M / 13F	0.2217
Age (years)	47.4 (+/-12.8)	49.6 (+/-11.4)	0.5465
White	12	21	0.5261
Afr Amer	0	4	0.2889
Hispanic	2	5	1.0000
Other Race	2	3	1.0000
BMI (kg/m^2^)	26.81 (+/-6.55)	36.58 (+/-11.02)	0.0005*
Hx of DM	0	3	0.5415
Tx for HTN	1	18	0.0014*
SBP (mmHg)	118.2 (+/-14.6)	130.3 (+/-11.4)	0.0034*
DBP (mmHg)	75.7 (+/-8.8)	82.9 (+/-9.2)	0.0129*
AHI-4%	0.59 (+/-0.60)	43.3 (+/-28.1)	<0.0001*
RDI (events/hr)	10.87 (+/-1.90)	58.74 (+/-4.88)	<0.0001*
Lowest O2 Sat %	91.7 (+/-2.5)	75.4 (+/-8.8)	<0.0001*
Sleep Efficiency %	81.1 (+/-2.9)	77.7 (+/-2.1)	0.3490
Stage N1%	7.96 (+/-1.59)	15.87 (+/-1.98)	0.0033*
Stage N2%	60.67 (+/-2.62)	57.68 (+/-2.29)	0.4240
Stage N3%	13.85 (+/-2.64)	6.84 (+/-1.44)	0.0146*
REM%	17.34 (+/-2.32)	14.48 (+/-1.92)	0.3724
Creatinine (mg/dl)	0.86 (+/-0.15)	0.87 (+/-0.19)	0.7956

*Significant at P<0.05

The control and OSA groups were otherwise similar in demographic composition, including in the proportions of men and women, the mean age, and by self-identified racial categorization. There was no significant difference in the number of diabetic patients between groups (0 versus 3 in control and OSA groups, respectively; p = 0.5415), or in the mean serum creatinine concentration between groups (control = 0.86 +/- 0.15 mg/dl, OSA = 0.87 +/- 0.19 mg/dl; p = 0.7956). (Table 1).

### Sleep Metrics

**Fig 1:**
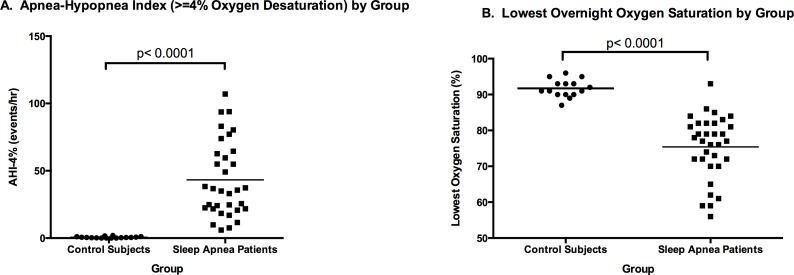
Sleep Apnea Severity between Control Subjects and Affected Patients. A depicts the individual apnea-hypopnea indices (average number of respiratory events with at least 4% oxygen desaturation per hour; AHI-4%) between control versus sleep apneic groups. B depicts the lowest percent oxygen saturation recorded by overnight pulse oximetry between groups. Horizontal lines within each group of data points indicate the group mean.

The difference between the mean AHI-4% of the OSA group versus the control group was highly statistically significant (43.3 +/- 28.1 events/hour versus 0.59 +/-0.60 events/hour, respectively; p<0.0001) (Fig 1A and Table 1). Similarly, the lowest percent oxygen saturation recorded by continuous pulse oximetry during PSG was significantly lower in the OSA group versus the control group (mean OSA = 75.4 +/- 8.8% versus mean Control = 91.7 +/- 2.5%; p<0.0001) (Fig 1B and Table 1). The RDI, which includes all scored respiratory events regardless of oxygen desaturation, was likewise highly different between groups (mean OSA = 58.74 +/- 4.88 events/hour versus mean Control = 10.87 +/- 1.90 events/hour; p<0.0001). Only the percentages of total sleep spent in non-rapid eye movement stages 1 (light sleep; N1%) and 3 (deep sleep; N3%) differed between OSA and control groups (mean OSA N1% = 15.87 +/- 1.98% versus mean Control N1% = 7.96 +/- 1.59%, p = 0.0033; mean OSA N3% = 6.84 +/- 1.44% versus mean Control N3% = 13.85 +/- 2.64%, p = 0.0146). The percentage of total sleep spent in rapid eye movement stage (REM%) was not different between groups (mean OSA REM% = 14.48 +/- 1.92 versus mean Control REM% = 17.34 +/- 2.32; p = 0.3724). (Table 1).

### Correlation between Mean Arterial Pressure and BMI

**Fig 2:**
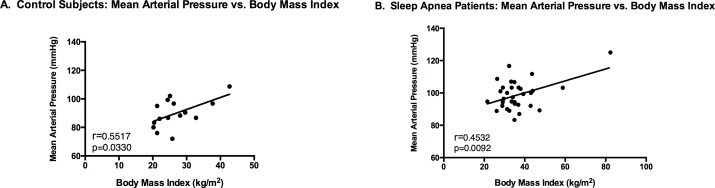
**Correlation between Mean Arterial Pressure and Body Mass Index by Group** Panel A depicts the individuals within the control group, and Panel B depicts the patients within the sleep apneic group.

For both the OSA and control groups, the mean arterial blood pressure (MAP) positively correlated with the BMI [r = 0.4532 (p = 0.0092) and r = 0.5517 (p = 0.0330), respectively] (Fig 2).

### Urinary Neutrophil Gelatinase-Associated Lipocalin Levels between OSA and Control Groups and Among Subset of OSA Patients before and after CPAP Therapy

**Fig 3:**
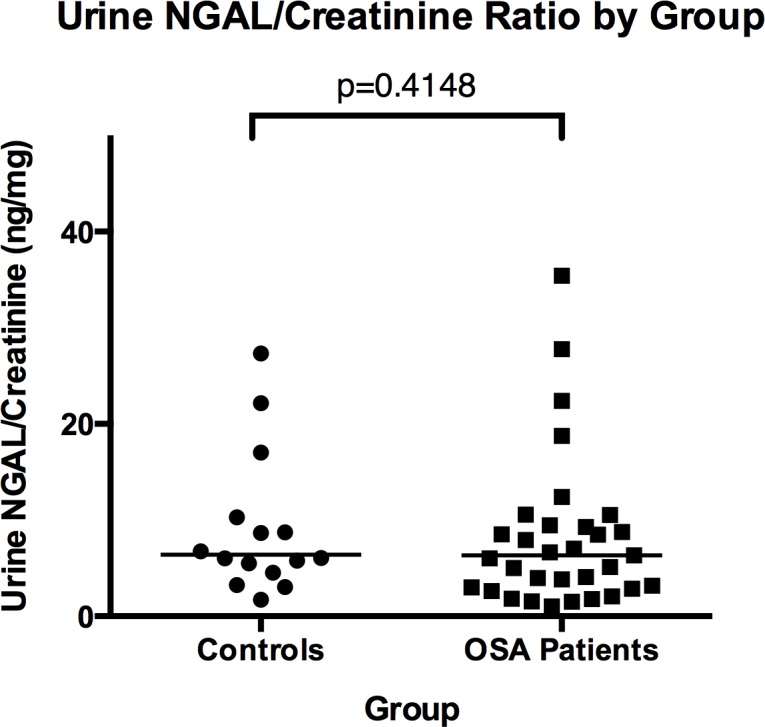
Individual Urinary NGAL-to-Creatinine Ratios (ng/mg) between Control versus Untreated Sleep Apneic (OSA) Groups. Horizontal lines within each group of data points indicate the group median.

**Fig 4:**
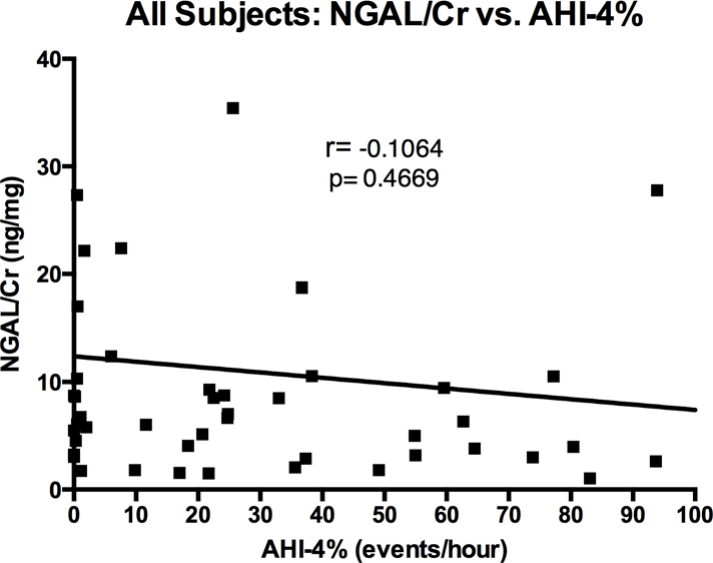
Individual NGAL-to-Creatinine Ratios (NGAL/Cr; ng/mg) versus Apnea-Hypopnea Indices (AHI-4%; events/hour) among All Subjects (Untreated Sleep Apnea and Control).

**Fig 5:**
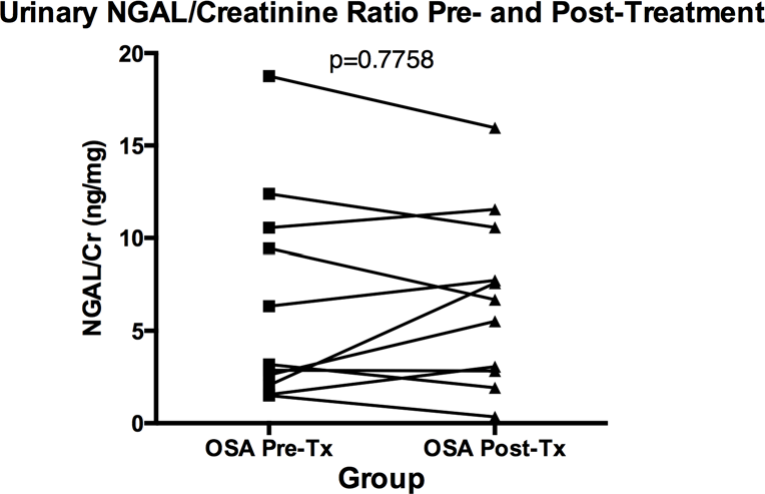
Urinary NGAL-to-Creatinine Ratios (NGAL/Cr; ng/mg) among 11 Sleep Apneic Patients (OSA) before (Pre-Tx) and after (Post-Tx) Successful Treatment with Continuous Positive Airway Pressure.

There was no significant difference between the urinary NGAL-to-creatinine ratios (ng NGAL/mg creatinine; NGAL/Cr) among the OSA patients as compared to the control subjects (median NGAL/Cr = 6.34 ng/mg versus 6.41 ng/mg, respectively; p = 0.4148) (Fig 3). There was no correlation between NGAL-to-creatinine ratio and AHI-4% (r = -0.1064, p = 0.4669) (Fig 4). Furthermore, among 11 OSA patients who submitted urine samples both before and after successful treatment with CPAP, treatment did not significantly affect the NGAL-to-creatinine ratio (Fig 5).

## Discussion

In this prospective case-control study comparing patients with severe, hypoxic OSA to unaffected but otherwise similar control subjects—all with normal serum creatinine—we found no difference between corrected urinary levels of the early renal tubular injury biomarker NGAL. Furthermore, successful CPAP therapy did not change these levels pre- and post-treatment among a subgroup of OSA patients. This current work appears to be the first to evaluate any well-established urinary renal tubular injury biomarker among OSA patients. Our hypothesis was that OSA’s recurrent hemodynamic events on the kidney—resembling renal ischemia-reperfusion injury [23,24]—would result in elevated urinary NGAL levels before any detectable increase in serum creatinine, as is observed in both experimental [25,26] and clinical [27–29] models of kidney ischemia-reperfusion injury. That we observed no elevation in corrected urinary NGAL levels (or in uncorrected levels; S1 Fig) among severe, untreated OSA patients was unexpected.

Our OSA group was representative of patients affected by this condition. For example, they had a significantly higher BMI and were much more likely to be treated for hypertension relative to controls. Moreover, their mean AHI-4% of greater than 40 events per hour classifies their OSA as severe. The OSA group further had evidence of sleep fragmentation, with double the percentage of time spent in light sleep (stage N1) and half the percentage of time spent in deep sleep (stage N3), as compared to controls. In other important respects, however, including gender distribution, age, self-identified race, diabetic status, and serum creatinine level, the OSA group was similar to control participants. Furthermore, the OSA group and the control group similarly demonstrated increasing MAP with increasing BMI, despite 18 of 33 members of the OSA group being treated with one or more antihypertensive medications.

Thus, our cohort appears sufficiently representative to detect a difference in urinary NGAL levels between groups if it existed. Furthermore, with 16 control participants and 33 untreated OSA patients, our power calculation revealed a >80% ability to detect a difference of at least 30% in NGAL/Cr between groups (at alpha = 0.05). Additionally, our pre- and post-CPAP results among a subgroup of successfully-treated OSA patients (with treatment strictly documented), in whom each patient acted as his/her own control, are consistent with no observed effect of OSA physiology on urinary NGAL levels. With regard to a potential difference in NGAL/Cr between OSA patients versus non-OSA subjects stratified by gender, we still found no effect of OSA: median female OSA versus non-OSA = 8.49 vs. 9.47 ng/mg (p = 0.5981 by Mann-Whitney test), and median male OSA versus non-OSA = 4.49 vs. 5.31 ng/mg (p = 0.9757 by Mann-Whitney test). Lastly, we found no correlation between urinary NGAL/Cr and the severity of OSA as indicated by the AHI-4%.

Since our data do not demonstrate an elevation of urinary NGAL among untreated sleep apneics with still-normal renal function (as per serum creatinine), we speculate that the kidney injury associated with OSA may be glomerular (as opposed to tubular) in nature. Indeed, microalbuminuria and frank proteinuria—the hallmarks of glomerular injury—have been observed in OSA patients by independent investigators [32–34]. Furthermore, glomerulomegaly and focal segmental glomerulosclerosis have been well-described on renal biopsy of OSA patients [35–37]. We must, however, acknowledge other possible conclusions that can be drawn from these data. It is possible, for example, that we were unable to detect a brief and/or low-magnitude increase in urinary NGAL based on our study design utilizing daytime urine collections. Furthermore, it may be that the renal insults of untreated OSA were sufficiently chronic among our study participants such that any increase in urinary NGAL eventually normalized over time.

Some additional limitations of our study require mention. First, our total cohort of 49 participants is not large. However, the controls and OSA patients were at such opposite poles of the sleep apnea spectrum that a difference in urinary NGAL levels, if one existed, should have emerged. Still, we cannot exclude the possibility that we were underpowered to detect a difference in urinary NGAL of less than 30% between groups. Second, it is possible that patients referred for evaluation of sleep complaints as a whole (regardless of OSA status and severity) have similarly elevated urinary NGAL levels as compared to subjects not experiencing sleep-related symptoms, which could obscure our ability to detect a difference between groups. A previous study designed to establish a normal reference range for urinary NGAL, derived from urine samples of healthy adults without CKD and using the same assay as we used, found a mean NGAL/Cr of 12.8 ng/mg and 25.0 ng/mg for men and women aged 41–50, respectively [38]. A separate study of non-healthy patients with cardiovascular disease (but normal pre-operative serum creatinine) undergoing cardiac surgery, also using the same NGAL assay, found a pre-operative urinary NGAL/Cr median value of 7.37 ng/mg (interquartile range = 3.11 to 22.24) among a group of 83 subjects [39]. Thus, considering our mean NGAL/Cr of 8.41 ng/mg among all male participants, mean of 13.77 ng/mg among all female participants, and median of 6.34 ng/mg among all 49 study participants (S1 Table), it does not appear sleep clinic patients as a whole have elevated NGAL/Cr. Third, since over 50% of the OSA patients were treated for hypertension, predominantly with an angiotensin-converting enzyme inhibitor (ACE-I) with or without diuretic, we considered the possibility that these medications may reduce urinary NGAL excretion and thereby obscure a difference between groups. However, review of the literature reveals that ACE-I and diuretics have not been shown to affect urinary NGAL levels [40,41]. Lastly, we evaluated only one renal tubular cell injury marker, although several others have been studied for the early detection of kidney injury, for example kidney injury molecule 1 (KIM-1) and *N*-Acetyl beta glucosaminidase (NAG) [42]. Nonetheless, review of the urinary biomarker literature reveals urinary NGAL performs as well, if not better, than these other biomarkers with regard to area under the receiver operating curve values in studies of renal ischemia-reperfusion injury, with values generally between 0.7 to 0.9 [25–29,42].

In conclusion, we prospectively studied a well-validated renal tubular epithelial injury molecule in the urine of OSA patients. The lack of urinary NGAL elevation among patients with severe OSA—and lack of change in these levels before and after CPAP therapy—that we observed potentially points away from kidney tubular injury as a mechanism of renal insult in OSA, within the limitations described. Further investigation is needed to clarify the mechanisms by which OSA may directly injury the kidneys.

## Supporting Information

S1 Fig Individual Uncorrected Urinary NGAL Levels (ng/ml) between Control versus Untreated Sleep Apneic (OSA) Groups. Horizontal lines within each group of data points indicate the group median.
(TIFF)

S1 Table NGAL/Cr Ratios by Gender and OSA Status. (DOCX)

## References

[1] Young T, Palta M, Dempsey J, Skatrud J, Weber S, Badr S. The occurrence of sleep-disordered breathing among middle-aged adults. N Engl J Med. 1993; 328(17): 1230–1235. 8464434 10.1056/NEJM199304293281704

[2] Peppard PE, Young T, Palta M, Skatrud J. Prospective study of the association between sleep-disordered breathing and hypertension. N Engl J Med. 2000; 342(19): 1378–1384. 10805822 10.1056/NEJM200005113421901

[3] Gottlieb DJ, Yenokyan G, Newman AB, O’Connor GT, Punjabi NM, Quan SF, et al Prospective study of obstructive sleep apnea and incident coronary heart disease and heart failure: the sleep heart health study. Circulation. 2010; 122(4): 352–360. 10.1161/CIRCULATIONAHA.109.901801 20625114 PMC3117288

[4] Yaggi HK, Concato J, Kernan WN, Lichtman JH, Brass LM, Mohsenin V. Obstructive sleep apnea as a risk factor for stroke and death. N Engl J Med. 2005; 353(19): 2034–2041. 16282178 10.1056/NEJMoa043104

[5] Ahmed SB, Ronksley PE, Hemmelgarn BR, Tsai WH, Manns BJ, Tonelli M, et al Nocturnal hypoxia and loss of kidney function. PLoS One. 2011; 6(4): e19029 10.1371/journal.pone.0019029 21559506 PMC3084745

[6] Sakaguchi Y, Hatta T, Hayashi T, Shoji T, Suzuki A, Tomida K. et al Association of nocturnal hypoxemia with progression of CKD. Clin J Am Soc Nephrol. 2013; 8(9): 1502–1507. 10.2215/CJN.11931112 23744006 PMC3805083

[7] Leong WB, Nolen M, Thomas GN, Adab P, Banerjee D, Taheri S. The impact of hypoxemia on nephropathy in extremely obese patients with type 2 diabetes mellitus. J Clin Sleep Med. 2014; 10(7): 773–778. 10.5664/jcsm.3870 25024655 PMC4067441

[8] Sakaguchi Y, Shoji T, Kawabata H, Niihata K, Suzuki A, Kaneko T, et al High prevalence of obstructive sleep apnea and its association with renal function among nondialysis chronic kidney disease patients in Japan: a cross-sectional study. Clin J Am Soc Nephrol. 2011; 6(5): 995–1000. 10.2215/CJN.08670910 21415314 PMC3087795

[9] Kato K, Takata Y, Usui Y, Shiina K, Asano K, Hashimura Y, et al Severe obstructive sleep apnea increases cystatin C in clinically latent renal dysfunction. Respir Med. 2011; 105(4): 643–649. 10.1016/j.rmed.2010.11.024 21183327

[10] Chou YT, Lee PH, Yang CT, Lin CL, Veasey S, Chuang LP, et al Obstructive sleep apnea: a stand-alone risk factor for chronic kidney disease. Nephrol Dial Transplant. 2011; 26(7): 2244–2250. 10.1093/ndt/gfq821 21317406

[11] Lee YC, Hung SY, Wang HK, Lin CW, Wang HH, Chen SW, et al Sleep apnea and the risk of chronic kidney disease: a nationwide population-based cohort study. Sleep. 2015; 38(2): 213–221. 10.5665/sleep.4400 25409108 PMC4288602

[12] Marcus JA, Pothineni A, Marcus CZ, Bisognano JD. The role of obesity and obstructive sleep apnea in the pathogenesis and treatment of resistant hypertension. Curr Hypertens Rep. 2014; 16(1): 411 10.1007/s11906-013-0411-y 24346827

[13] Nannapaneni S, Ramar K, Surani S. Effect of obstructive sleep apnea on type 2 diabetes mellitus: A comprehensive literature review. World J Diabetes. 2013; 4(6): 238–244. 10.4239/wjd.v4.i6.238 24379913 PMC3874482

[14] Somers VK, Dyken ME, Clary MP, Abboud FM. Sympathetic neural mechanisms in obstructive sleep apnea. J Clin Invest. 1995; 96(4): 1897–1904. 7560081 10.1172/JCI118235 PMC185826

[15] Gilmartin GS, Lynch M, Tamisier R, Weiss JW. Chronic intermittent hypoxia in humans during 28 nights results in blood pressure elevation and increased muscle sympathetic nerve activity. Am J Physiol Heart Circ Physiol. 2010; 299(3): H925–931. 10.1152/ajpheart.00253.2009 20581089 PMC4116417

[16] Fletcher EC, Bao G, Li R. Renin activity and blood pressure in response to chronic episodic hypoxia. Hypertension. 1999; 34(2): 309–314. 10454459 10.1161/01.hyp.34.2.309

[17] Nicholl DD, Hanly PJ, Poulin MJ, Handley GB, Hemmelgarn BR, Sola DY, et al Evaluation of continuous positive airway pressure therapy on renin-angiotensin system activity in obstructive sleep apnea. Am J Respir Crit Care Med. 2014; 190(5): 572–580. 10.1164/rccm.201403-0526OC 25033250

[18] Yamauchi M, Kimura H. Oxidative stress in obstructive sleep apnea: putative pathways to the cardiovascular complications. Antioxid Redox Signal. 2008; 10(4): 755–768. 10.1089/ars.2007.1946 18177236

[19] Somers VK, White DP, Amin R, Abraham WT, Costa F, Culebras A, et al Sleep apnea and cardiovascular disease: an American Heart Association/American College Of Cardiology Foundation Scientific Statement from the American Heart Association Council for High Blood Pressure Research Professional Education Committee, Council on Clinical Cardiology, Stroke Council, and Council On Cardiovascular Nursing. In collaboration with the National Heart, Lung, and Blood Institute National Center on Sleep Disorders Research (National Institutes of Health). Circulation. 2008; 118(10): 1080–1111. 10.1161/CIRCULATIONAHA.107.189375 18725495

[20] Ryan S, McNicholas WT. Intermittent hypoxia and activation of inflammatory molecular pathways in OSAS. Arch Physiol Biochem. 2008; 114(4): 261–266. 10.1080/13813450802307337 18946786

[21] Bruno RM, Rossi L, Fabbrini M, Duranti E, Di Coscio E, Maestri M, et al Renal vasodilating capacity and endothelial function are impaired in patients with obstructive sleep apnea syndrome and no traditional cardiovascular risk factors. J Hypertens. 2013; 31(7): 1456–1464; discussion 1464. 10.1097/HJH.0b013e328360f773 23965549

[22] Kinebuchi S, Kazama JJ, Satoh M, Sakai K, Nakayama H, Yoshizawa H, et al Short-term use of continuous positive airway pressure ameliorates glomerular hyperfiltration in patients with obstructive sleep apnoea syndrome. Clin Sci (Lond). 2004; 107(3): 317–322. 15191364 10.1042/CS20040074

[23] Linz D, Mahfoud F, Linz B, Hohl M, Schirmer SH, Wirth KJ, et al Effect of obstructive respiratory events on blood pressure and renal perfusion in a pig model for sleep apnea. Am J Hypertens. 2014; 27(10): 1293–1300. 10.1093/ajh/hpu036 24622919

[24] Eltzschig HK, Eckle T. Ischemia and reperfusion—from mechanism to translation. Nat Med. 2011; 17(11): 1391–1401. 10.1038/nm.2507 22064429 PMC3886192

[25] Singer E, Marko L, Paragas N, Barasch J, Dragun D, Muller DN, et al Neutrophil gelatinase-associated lipocalin: pathophysiology and clinical applications. Acta Physiol (Oxf). 2013; 207(4): 663–672. 23375078 10.1111/apha.12054 PMC3979296

[26] Mishra J, Ma Q, Prada A, Mitsnefes M, Zahedi K, Yang J, et al Identification of neutrophil gelatinase-associated lipocalin as a novel early urinary biomarker for ischemic renal injury. J Am Soc Nephrol. 2003; 14(10): 2534–2543. 14514731 10.1097/01.asn.0000088027.54400.c6

[27] Parikh CR, Coca SG, Thiessen-Philbrook H, Shlipak MG, Koyner JL, Wang Z, et al Postoperative biomarkers predict acute kidney injury and poor outcomes after adult cardiac surgery. J Am Soc Nephrol. 2011; 22(9): 1748–1757. 10.1681/ASN.2010121302 21836143 PMC3171945

[28] Koyner JL, Vaidya VS, Bennett MR, Ma Q, Worcester E, Akhter SA, et al Urinary biomarkers in the clinical prognosis and early detection of acute kidney injury. Clin J Am Soc Nephrol. 2010; 5(12): 2154–2165. 10.2215/CJN.00740110 20798258 PMC2994075

[29] Wagener G, Jan M, Kim M, Mori K, Barasch JM, Sladen RN, et al Association between increases in urinary neutrophil gelatinase-associated lipocalin and acute renal dysfunction after adult cardiac surgery. Anesthesiology. 2006; 105(3): 485–491. 16931980 10.1097/00000542-200609000-00011

[30] K/DOQI clinical practice guidelines for chronic kidney disease: evaluation, classification, and stratification. Am J Kidney Dis. 2002; 39(2 Suppl 1): S1–266. 11904577

[31] Iber C, Ancoli-Israel S, Chesson A, Quan SF for the American Academy of Sleep Medicine. The AASM Manual for the Scoring of Sleep and Associated Events: Rules, Terminology and Technical Specifications. 1st ed. Westchester, IL: American Academy of Sleep Medicine; 2007.

[32] Chaudhary BA, Sklar AH, Chaudhary TK, Kolbeck RC, Speir WA Jr. Sleep apnea, proteinuria, and nephrotic syndrome. Sleep. 1988; 11(1): 69–74. 3363272 10.1093/sleep/11.1.69

[33] Faulx MD, Storfer-Isser A, Kirchner HL, Jenny NS, Tracy RP, Redline S. Obstructive sleep apnea is associated with increased urinary albumin excretion. Sleep. 2007; 30(7): 923–929. 17682664 10.1093/sleep/30.7.923 PMC1978377

[34] Tsioufis C, Thomopoulos C, Dimitriadis K, Amfilochiou A, Tsiachris D, Selima M, et al Association of obstructive sleep apnea with urinary albumin excretion in essential hypertension: a cross-sectional study. Am J Kidney Dis. 2008; 52(2): 285–293. 10.1053/j.ajkd.2008.05.001 18617307

[35] Serra A, Romero R, Lopez D, Navarro M, Esteve A, Perez N, et al Renal injury in the extremely obese patients with normal renal function. Kidney Int. 2008; 73(8): 947–955. 10.1038/sj.ki.5002796 18216780

[36] Jennette JC, Charles L, Grubb W. Glomerulomegaly and focal segmental glomerulosclerosis associated with obesity and sleep-apnea syndrome. Am J Kidney Dis. 1987; 10(6): 470–472. 3687938 10.1016/s0272-6386(87)80196-8

[37] Bailey RR, Lynn KL, Burry AF, Drennan C. Proteinuria, glomerulomegaly and focal glomerulosclerosis in a grossly obese man with obstructive sleep apnea syndrome. Aust N Z J Med. 1989; 19(5): 473–474. 2590100 10.1111/j.1445-5994.1989.tb00310.x

[38] Pennemans V, Rigo JM, Faes C, Reynders C, Penders J, Swennen Q. Establishment of reference values for novel urinary biomarkers for renal damage in the healthy population: are age and gender an issue? Clin Chem Lab Med. 2013; 51(9): 1795–1802. 10.1515/cclm-2013-0157 23648635

[39] Liu S, Che M, Xue S, Xie B, Zhu M, Lu R, et al Urinary L-FABP and its combination with urinary NGAL in early diagnosis of acute kidney injury after cardiac surgery in adult patients. Biomarkers. 2013; 18(1): 95–101. 10.3109/1354750X.2012.740687 23167703

[40] Nielsen SE, Schjoedt KJ, Astrup AS, Tarnow L, Lajer M, Hansen PR, et al Neutrophil Gelatinase-Associated Lipocalin (NGAL) and Kidney Injury Molecule 1 (KIM1) in patients with diabetic nephropathy: a cross-sectional study and the effects of lisinopril. Diabet Med. 2010; 27(10): 1144–1150. 10.1111/j.1464-5491.2010.03083.x 20854382

[41] Damman K, Kam Ng Chuen MJ, MacFadyen RJ, Lip GY, Gaze D, Collinson PO, et al Volume status and diuretic therapy in systolic heart failure and the detection of early abnormalities in renal and tubular function. J Am Coll Cardiol. 2011; 57(22): 2233–2241. 10.1016/j.jacc.2010.10.065 21616283

[42] Vanmassenhove J, Vanholder R, Nagler E, Van Biesen W. Urinary and serum biomarkers for the diagnosis of acute kidney injury: an in-depth review of the literature. Nephrol Dial Transplant. 2013; 28(2): 254–273. 10.1093/ndt/gfs380 23115326

