# Supplementary figures and images for: Urinary Neutrophil Gelatinase-Associated Lipocalin (NGAL) in Patients with Obstructive Sleep Apnea
Source: PLoS One. 2016 May 5;11(5):e0154503. doi: 10.1371/journal.pone.0154503 (PMC4858300; doi:10.1371/journal.pone.0154503)

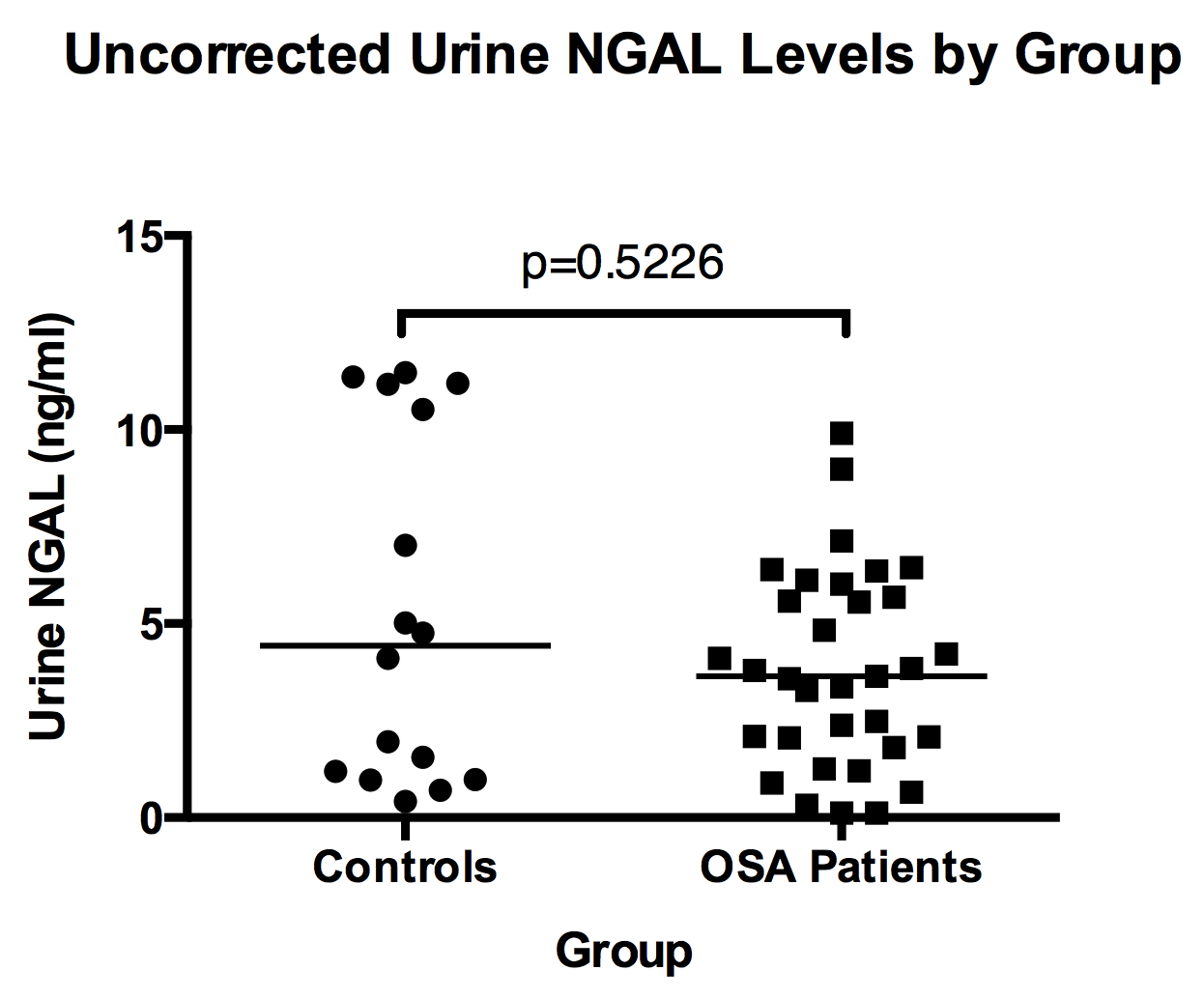

Supplement: S1 Fig — Horizontal lines within each group of data points indicate the group median. (TIFF) [file pone.0154503.s001.tiff]
