# Supplementary material for: Urinary Neutrophil Gelatinase-Associated Lipocalin (NGAL) in Patients with Obstructive Sleep Apnea
Source: PLoS One. 2016 May 5;11(5):e0154503. doi: 10.1371/journal.pone.0154503 (PMC4858300; doi:10.1371/journal.pone.0154503)
Supplement: S1 Table — (DOCX) [file pone.0154503.s002.docx]

| **Gender (N)** | **Mean**  **NGAL/Cr (ng/mg)** | **Median**  **NGAL/Cr**  **(ng/mg)** |
| --- | --- | --- |
| All Men (26) | 8.41 (+/-13.73) | 4.78 (2.41, 8.57) |
| OSA Men (20) | 9.39 (+/-15.56) | 4.49 (2.01, 9.10) |
| Control Men (6) | 5.14 (+/-2.56) | 5.31 (2.71, 7.25) |
| All Women (23) | 13.77 (+/-14.73) | 8.64 (5.79, 17.02) |
| OSA Women (13) | 10.98 (+/-9.74) | 8.49 (5.05, 11.46) |
| Control Women (10) | 17.39 (+/-19.44) | 9.47 (5.71, 23.44) |
| All Subjects (49) | 10.93 (+/-14.31) | 6.34 (3.21, 10.42) |

Table shows mean (+/- standard deviation), median (25%ile, 75%ile), or N=number of patients falling into category
